# An Eigenvalues-Based Covariance Matrix Bootstrap Model Integrated With Support Vector Machines for Multichannel EEG Signals Analysis

**DOI:** 10.3389/fninf.2021.808339

**Published:** 2022-02-03

**Authors:** Hanan Al-Hadeethi, Shahab Abdulla, Mohammed Diykh, Ravinesh C. Deo, Jonathan H. Green

**Affiliations:** ^1^School of Mathematics Physics and Computing, University of Southern Queensland, Toowoomba, QLD, Australia; ^2^USQ College, University of Southern Queensland, Toowoomba, QLD, Australia; ^3^Information and Communication Technology Research Group, Scientific Research Centre, Al-Ayen University, Nasiriyah, Iraq; ^4^School of Sciences, University of Southern Queensland, Toowoomba, QLD, Australia; ^5^College of Education for Pure Science, University of Thi-Qar, Nasiriyah, Iraq; ^6^Faculty of the Humanities, University of the Free State, Bloemfontein, South Africa

**Keywords:** alcoholism, electroencephalogram, covariance matrix, support vector machine (SVM), eigenvalues and fruit fly optimization

## Abstract

Identification of alcoholism is clinically important because of the way it affects the operation of the brain. Alcoholics are more vulnerable to health issues, such as immune disorders, high blood pressure, brain anomalies, and heart problems. These health issues are also a significant cost to national health systems. To help health professionals to diagnose the disease with a high rate of accuracy, there is an urgent need to create accurate and automated diagnosis systems capable of classifying human bio-signals. In this study, an automatic system, denoted as (CT-BS- Cov-Eig based FOA-F-SVM), has been proposed to detect the prevalence and health effects of alcoholism from multichannel electroencephalogram (EEG) signals. The EEG signals are segmented into small intervals, with each segment passed to a clustering technique-based bootstrap (CT-BS) for the selection of modeling samples. A covariance matrix method with its eigenvalues (Cov-Eig) is integrated with the CT-BS system and applied for useful feature extraction related to alcoholism. To select the most relevant features, a nonparametric approach is adopted, and to classify the extracted features, a radius-margin-based support vector machine (F-SVM) with a fruit fly optimization algorithm (FOA), (i.e., FOA-F-SVM) is utilized. To assess the performance of the proposed CT-BS model, different types of evaluation methods are employed, and the proposed model is compared with the state-of-the-art models to benchmark the overall effectiveness of the newly designed system for EEG signals. The results in this study show that the proposed CT-BS model is more effective than the other commonly used methods and yields a high accuracy rate of 99%. In comparison with the state-of-the-art algorithms tested on identical databases describing the capability of the newly proposed FOA-F-SVM method, the study ascertains the proposed model as a promising medical diagnostic tool with potential implementation in automated alcoholism detection systems used by clinicians and other health practitioners. The proposed model, adopted as an expert system where EEG data could be classified through advanced pattern recognition techniques, can assist neurologists and other health professionals in the accurate and reliable diagnosis and treatment decisions related to alcoholism.

## Introduction

The human brain, as an integral part of the central nervous system (CNS), operates normally by receiving signals from the body’s organs and providing information to the muscles (Pelvig et al., 2008). The effects of alcohol on the CNS can lead to long- and short-term issues such as impaired vision, impaired hearing, dementia, and depression (Deiner and Silverstein, 2009). Alcoholism is a common neurological disorder caused by excessive and repetitive drinking of alcoholic beverages; the harmful effects of alcoholic beverages could be physical and mental as well as social, legal, and economic (Lieber, 1995; Volkow et al., 2017). The heavy consumption of alcohol disturbs the functioning of the entire nervous system, especially the brain. It not only weakens the brain neurons but also leads to cognitive and mobility weakness (Knight and Longmore, 1994; Oscar-Berman et al., 1997). Based on the latest reports issued by the WHO https://www.who.int/health-topics/alcohol#tab=tab_1, three million deaths every year are caused by the harmful use of alcohol. In addition, more than 200 disease- and injury-related conditions are caused by the excessive use of alcohol. An effective method of recognizing alcoholics from nonalcoholics could decrease unnecessary economic losses and social problems as well as expedite diagnosis in clinical settings.

Electroencephalogram (EEG) technology is becoming increasingly important in the identification, diagnosis, and treatment of mental and neurodegenerative diseases and abnormalities (Isaksson et al., 1981). The function of the EEG assists physicians in establishing an accurate diagnosis. Thus, it can be utilized as a diagnostic tool to discern alcoholics from nonalcoholic subjects based on the variation in the signals.

Much effort has been expended in deducing the preferred classification method in analyzing EEG signals for alcoholism. For instance, Faust et al. (2008) analyzed normal, epileptic, and alcoholic EEG signals utilizing fast Fourier transform (FFT) and autoregressive (AR) model and their techniques. Their results showed that the power spectral density (PSD) of these signals was varied. Patidar et al. (2017) applied tunable Q-wavelet transform (TQWT) to decompose EEG rhythms into different bands. The principal component analysis (PCA) was utilized for feature extraction and then fed to a least squares-support-vector machine (LS-SVM). Cao et al. (2017) utilized a synchronization likelihood to measure synchronization variations among 28 alcoholics and 28 control subjects. The study showed that the synchronization for the control group reflected the complexity levels of the cognitive tasks, while the alcoholics only displayed erratic changes. Lin et al. (2009) analyzed the clinical alcoholic and normal control FP1 EEG signals based on a Hilbert-Huang Transformation. The PCA and WT were also applied to analyze EEG data by Sun et al. (2006), and other studies have used the power spectrum of Haar mother wavelet, approximate entropy, sample entropy, and empirical mode decomposition. Kousarrizi et al. (2009) applied the power spectrum of the Haar mother wavelet to extract the features with PCA. The extracted features were fed to a support vector machine (SVM) and neural networks. The simulation results showed that their method achieved a higher rate of classification accuracy than other methods. Shooshtari and Setarehdan (2010) proposed a reduction method to select an optimum subset of EEG channels based on spectral analysis and correlation matrices: their technique was successful in selecting an optimal number of channels. Kumar et al. (2012) employed an approximate entropy and sample entropy to extract entropy features from EEG time series: they illustrated that the average value of ApEn and SampEn for an epileptic time series was less than that of a nonepileptic time series. The study of Priya et al. (2018) has used mode decomposition (EMD) for features extraction.

Time-frequency (T–F) image information, high-pass infinite impulse response (IIR) filter with zero phase distortion, Separability and Correlation analysis (SEPCOR), computer-aided diagnosis, and EEG rhythms-based features were utilized in many studies that follow. Bajaj et al. (2017) proposed a new hybrid method to classify automatically an alcoholic and a control EEG signal based on T–F image information and found it useful in conveying key characteristics in EEG signals. The results of this study were promising. Fattah et al. (2015) proposed a new method based on a high-pass IIR filter with zero phase distortion, which aimed to preserve the Gamma band and all higher frequencies with K-nearest neighbor (KNN) classifier and leave-one-out cross-validation technique. Their proposed scheme also classified alcoholic and nonalcoholic subjects with a higher rate of accuracy than did existing methods. To select an optimal feature subset automatically and to obtain a minimum correlation between selected channels and maximum class separation, a statistical feature selection technique based on SEPCOR was proposed by Shri and Sriraam (2016); a significant improvement in the classification accuracy based on the SEPCOR method was noted in that study compared with feature selection methods used in previous studies. The study of Acharya et al. (2014) presented a review of the known features of EEGs gained from people with alcoholism. EEG-rhythms-based features for automatic identification of alcohol EEG signals were also proposed by the study of Taran and Bajaj (2017); in that study, an extreme learning machine (ELM) and a least squares SVM classifiers were used to detect nonalcoholic and alcoholic EEG signals, with the investigators’ techniques showing an accuracy of 97.92%.

Recently, there is a trend of using deep learning models for BP estimation; for example, Gao et al. (2021) designed an approach that combined recurrence plots and convolutional neural network to recognize fatigue driving. They showed that that complex network based on a deep learning model gave a high recognition rate. Tao et al. (2020) developed an attention-based convolutional recurrent neural network mode to classify emotion EEG signals. In that study, the convolutional recurrent neural network was used to extract spatial characteristics of EEG signals. Singhal et al. (2021) integrated FFT, a convolution neural network, and long short-term memory to classify EEG recordings into an alcoholic or control. Buriro et al. (2021) utilized wavelet scattering transform with a convolutional neural network and SVM to classify alcoholism from EEG signals. They found that wavelet scattering transform-based features with a conventional neural network had a high potential to detect alcoholic subjects.

As demonstrated in previous studies, finding new techniques for the detection of alcoholism can help in further clinical applications and research. The present study provides a new mechanism for the classification of alcoholism from multichannel EEG signals. This study has developed a new machine learning model for the reduction of data prior to the classification process by integrating the clustering and bootstrapping clustering technique-based bootstrap (CT-BS) technique in one phase of model design. To detect and further analyze the abnormalities in the EEG signal, the eigenvalues of the covariance matrix, determined from EEG signals, are investigated using a statistical method by extracting ten statistical features from the eigenvalues of the covariance matrix. These features are represented by the *mean, median, maximum, minimum, mode, range, SD, variation, skewness*, and *kurtosis* commonly used in EEG classification problems. To improve the automated detection system, a combination-based approach using the F-SVM and fruit fly optimization algorithm (FOA), i.e., FOA-F-SVM, has been proposed to correctly classify alcoholism from multichannel EEG signals. Based on an extensive literature search, the CT-BS- covariance matrix method with its eigenvalues (Cov-Eig)-based FOA-F-SVM model is proposed in this study for the first time to analyze and detect alcoholism from EEG signals. In respect to the results, compared with the other algorithms, the proposed model, CT-BS-Cov-Eig-based FOA-F-SVM, has promising performance, and can, therefore, be adopted as a classification technique for alcoholism-detection in EEG signals.

This research article is divided into several sections: Section 2 presents the methodology; Section 3 contains a description and explanation of the datasets, segmentation, sampling, feature extraction, and feature selection; Section 4 contains performance evaluation methods; Section 5 includes radius-margin-based SVM (F-SVM), fruit fly optimization algorithm (FOR), and the proposed classification model FOR-F-SVM; Section 6 includes experimental results, evaluation of the performance of the proposed FOA-F-SVM model, channels selection based on classification accuracy, comparison of classification accuracy of the proposed model FOA-F-SVM with KNN, k-means, and SVM, and comparison the proposed model, FOA-F-SVM, with previous studies and discussion; and Section 7 presents the conclusions.

## Materials and Methods

### Experimental Effects of Alcoholism From Multichannel Electroencephalogram Dataset

In the work described in this study, we have utilized a public database known as the machine learning repository (UCI) Knowledge Discovery in Databases (KDD) Archive www.kdd.ics.usi.edu from Irvine, CA: the University of California, Department of Information and Computer Science (Hettich and Bay, 1999). Data were collected from 122 participants; for each participant, there were 120 trials with three kinds of stimuli (Zhang et al., 1997). The EEG signals were recorded from 64 channels, two electrooculography (EOG) channels, and one reference electrode. The duration of each trial was one second and the sampling rate of all channel data was 256 Hz. UCI KDD contains three types of datasets, which are SMNI CMI TEST, SMNI CMI TRAIN, and FULL, respectively. FULL datasets contain a few all-zero recordings (Zhu et al., 2011); therefore, the first two databases were utilized. There are 600 recorded files in SMNI CMI TEST and the same number in the SMNI CMI TRAIN, which equals 1,200 recorded files, and for each recording, there are signals from 64 electrode caps.

### Methodology

**FIGURE 1:**
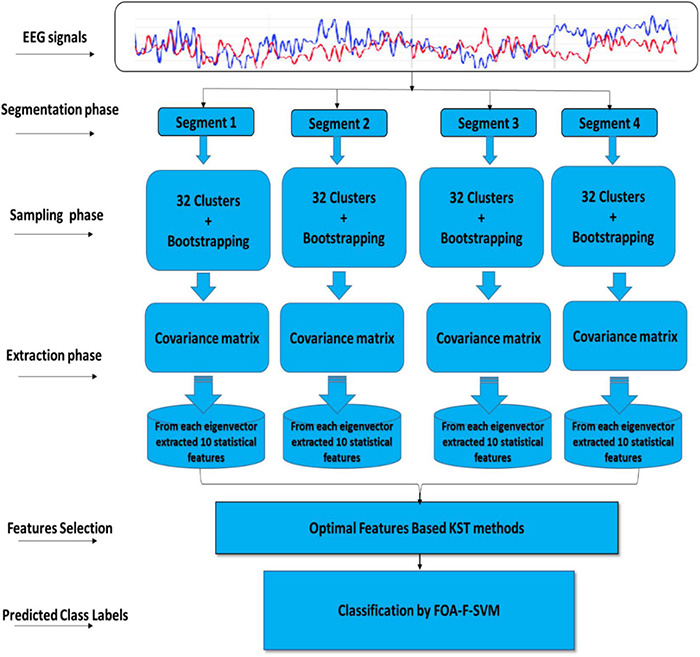
A flow diagram representation of the algorithm developed for detection and classification of alcoholism-based EEG signals.

This article describes the design of a new technique trained to classify alcoholism from multichannel EEG signals. A hybrid method called (CT-BS) by integrating clustering technique (CT) and bootstrapping (BS) has been developed to reduce the dimensions of the EEG data. Then, the covariance matrix with its eigenvalues, coupled with the FOA-F-SVM, is proposed to predict alcoholism in patients’ recordings. KDD recorded at the University of California, Department of Information and Computer Science (Gao et al., 2021) is used for the evaluation of the proposed model. Figure 1 demonstrates the proposed model. The EEG signals are divided into four segments; after that, each segment is sent into the CT-BS method for the sampling phase. To extract EEG features, the covariance matrix with its eigenvalues is applied. Following this, to detect and analyze abnormalities in the EEG signal, the eigenvalues of the covariance matrix are investigated and ten statistical features were extracted from eigenvalues of each covariance matrix. These features are *mean, median, maximum, minimum, mode, range, SD, variation, skewness*, and *kurtosis*. In this study, we used a nonparametric method, named the Kolmogorov–Smirnov test (KST), for selecting the most relevant features. The selected features are fed to the FOA-F-SVM to classify EEG signals. To estimate the performance of the proposed model, different types of assessment metrics, such as accuracy, sensitivity, and specificity, are used in the performance evaluation.

#### Segmentation

**FIGURE 2:**
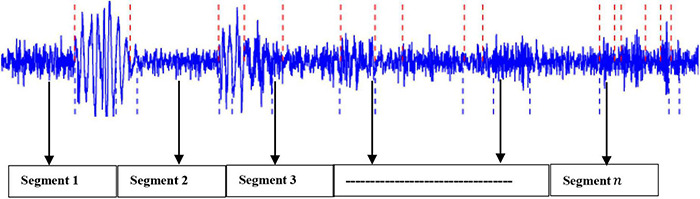
An example of EEG being partitioned into segments.

Based on our previous work (Diykh et al., 2018, 2019a,2019b,^2020^, ^2021^), this project has applied the sliding window technique to split the EEG signals into their respective periods. It was found that the proposed method generated highly satisfactory classification accuracy. Mathematically, let an EEG signal be denoted as: *X* = *x*_1_,*x*_2_,…..,*x*_*n*_ with *n* being the data points. In this study, the EEG signal *X* was segmented into *m* segments, with each segment containing *k* datapoints (Diykh et al., 2020, 2021). Figure 2 shows an example of an EEG signal being partitioned into segments.

#### Clustering Technique Coupled With-Based Bootstrap

To design a powerful sampling technique, a hybrid method that integrates the CT and BS, (CT-BS), is proposed in this study for reducing the dimensionality of EEG signals. This also prevents problems such as bias and variation that may occur when applying a CT. Not only is BS a method that depends on random sampling with replacement, but it also estimates the properties of an estimator. Adapting standard errors for clustering can be a very important part of any statistical analysis (Hennig, 2007); further, in terms of statistical modeling, validation is extremely important in cluster analysis because CTs resort to generate clustering even for completely homogeneous data groups. Most CTs suppose a certain paradigm for clusters, and this could be adequate for some portions of data, but not for others. The issue of stability in cluster analysis is complex, but it is considered an important part of cluster validity (Alonso et al., 2007). We propose to use the bootstrap method to reduce the error rate, which leads to reducing the bias and variation. The main concept behind utilizing the nonparametric bootstrap for the estimation of cluster constancy or stability is the following:

Suppose that there is a mixture distribution $K=\sum_{i=1}^{z}\varepsilon_{i}K_{i}$ where *i = 1, 2, 3, …, z*, are the distributions generating *z* “true” clusters, and ε_*i*_ is the probability that a point from *K_i* is drawn (Hira and Gillies, 2015). For a given dataset with *n* points, the “true” clustering would then be composed of *z* clusters, each of which includes precisely the points generated by *K_*i*_, i = 1, 2, 3,…, z*. The dataset, when generated from *K*, is clustered; the generated clusters vary from the “true”’ clusters because the clustering approach introduces an assured bias and variation.

The concept of bias and variation can be expressed *via* the maximum *Jaccard* coefficient. It is a measure of similarity for the two sets of data, with a range from 0 to 100%. A high percentage refers that two populations are similar among all the points created *via K*_*i*_ and the two sets belong to an identical cluster. The bootstrap is habitually utilized to grant an idea of bias and variation caused *via* a certain statistical approach because no true clustering is known and there is no true underlying distribution. To simulate *K*, the empirical distribution of the observed dataset is taken. The originally found clusters can be treated as the “true” ones, and the points can be drawn from the dataset. The mean maximal *Jaccard* coefficient can be explained as denoting the stability of the authentic clusters. Given a number *b* of bootstrap replications and a cluster *C* from the original clustering *E_*n*_(y)*, the schema works as below:

Reiterate for *i = 1, 2, 3, …, b*:

• For *n* points, draw a bootstrap sample $y_{n}^{i}$ with replacement from the original dataset *y_n*.
• Calculate the clustering En(y)in.
• Suppose $y_{*}^{i}=y_{n}\cap y_{n}^{i}$ be the points of the original dataset that are also in the bootstrap sample. Suppose C=i*C∩yni, $△=E_{n}\left(y_{n}^{i}\right)\cap y_{*}^{i}$.
• If $C_{*}^{i}\neq \emptyset$, calculate the maximum *Jaccard* similarity between the induced cluster $C_{*}^{i}$ and the induced new clustering △ on $y_{*}^{i}$:$\tau_{C,i}=max_{D\in △}\tau \left(C_{*}^{i},D\right)$ (i.e., *D* is the maximizer of $\tau \left(C_{*}^{i},D\right)$; else τ=C,i0).
  where *Jaccard* coefficient (Jaccard, 1901): $\tau \left(C,D\right)=\frac{\left|C\cap D\right|}{\left|C\cup D\right|},C,D\subseteq y_{n}$.

This generates a sequence τ_(*C*,*i*)_, *i = 1, 2, 3, …,b*. Based on (Cameron et al., 2008; Diykh et al., 2019b) they suggested the mean: $\tau_{C}=\frac{1}{b^{*}}\sum_{i=1}^{b}\tau_{\left(C,i\right)}$ as stability measure (b* being the number of bootstrap replications for which $C_{*}^{i}\neq \emptyset$ and is utilised here because in all other cases τ_(*C*,*i*)_ = 0).

#### Features Extraction

In machine learning, with huge dimensions of data, the necessity to provide a reliable analysis grows exponentially (Alonso et al., 2007; Hira and Gillies, 2015). There are diverse types of mental and neurological conditions where the EEG data size is huge and requires observation by the clinician over an extended period. Alcoholism EEG signals may contain valuable and useful information about the different states of the brain. Since the biological signal is highly random in both the time and frequency domain, computerized analysis is indispensable. Due to the signals being nonstationary, appropriate analysis is fundamental for EEG to differentiate the alcoholic/control EEG signals. A covariance matrix method that was used in previous work (Al-Hadeethi et al., 2020) is proposed to reduce the EEG signal (and data) dimensionality while extracting the most important features for better classification accuracy.

The time series (EEG signals) can be defined as a vector of length *X* = {*x*_1_,*x*_2_,…..,*x*_*n*_}. Feature nominees can be integrated into a feature vector for a point in time series. Let *P*_*i*_ the number of features. The feature vector for the Nth point of the subsequence can be manifested as (Ergezer and Leblebicioǧlu, 2016, 2018):


(4.1) $$h_{N}=\left[P_{N1},P_{N2},\ldots ,P_{NQ}\right]$$

After combining the feature vectors for all points, this study gets a feature matrix *H*,


(4.2) $$H=\left[\begin{array}{c} \begin{array}{ccc} P_{11} & \cdots & P_{1Q} \end{array}\\ \vdots \\ \begin{array}{ccc} P_{W1} & \cdots & P_{WQ} \end{array} \end{array}\right]$$

It can be calculated as the covariance of the feature matrix as follows:


(4.3) $$COV=\frac{1}{W-1}\sum_{i=1}^{W-1}\left(H_{i}-\mu \right){\left(H_{i}-\mu \right)}^{T}$$

where μ is the mean vector of feature vectors {*h*_1_,*h*_2_,…,*h*_*W*_}.

Based on separating the time series into *L* overlapping subsequences with each having a length *W*, the general representation was adapted for the time series classification problem. In this study, to decrease the dimensionality of data which leads to enhance detection of possible abnormalities in the prescribed EEG signal, the eigenvalues of the covariance matrix are investigated by extracting 10 features from each eigenvector.

In this research, the data were derived from multichannel EEG signals, where each channel consists of a matrix (256 × 30), where 256 represents the number of rows and 30 represents the number of columns. For more clarification, we will explain using the following example: an experiment of 61channels that consists of a matrix (15,616 × 30) was used in this article. The time series was divided into four segments (*n* = 4), each segment containing (3,904 × 30) data points. Then, each segment of 3,904 datapoints was divided into 32 clusters with each cluster containing 120 data points. Based on our previous work (Zhu et al., 2011), it was found that dividing each EEG segment into 32 clusters gave satisfactory results. As a result, each segment was represented by a matrix of 120 × 32. To reduce the dimensionality of each segment, the sampling technique was applied to reduce the number of clusters. The number of clusters was reduced from 32 to 30. Consequently, each segment was represented by 120 × 30 instead of 120 × 32. To remove any redundant information and extract features from each cluster, each cluster was divided into 4 sub-clusters, and a covariance matrix was applied to each subcluster, from each its eigenvector, 10 statistical features were selected to form a vector of 40 statistical features. As a result, each segment was represented by a matrix of 40 × 30, where 40 refers to the number of features and 30 indicates the number of clusters.

#### Feature Selection

In the work described in this study, one of the primary objectives of conducting many experiments was to find the optimal features that improved results. The features briefly summarize the most important information in the data, thus, this is used in cases where there is a large number of dimensions (Abdulla et al., 2019). Selecting the optimal features could lead to a high rate of classification accuracy. Therefore, six experiments were conducted on EEG channels to determine the features set using KST. More details are given in the results section.

## Classification Approach Based on SVM

### Radius-Margin-Based Support Vector Machine

Given the training set *q* = (*x*_1_,*y*_1_),(*x*_*n*_,*y*_*n*_), the fundamental SVM paradigm is displayed below. The paradigm only deems the maximization of margin. However, an accurate description can explain that the generalization error bounds of SVM are the function of radius and margin (Hedges et al., 1999).


(4.4) |d,b,δmin21|(n)||+22Z∑iδi

$$s.t.y_{i}\left(n^{T}x_{i}b\right)\geq 1-\delta_{i}\forall_{i}$$

$$\delta_{i}=0,i=1,2,3,\ldots ,$$

Given the radius, a group of researchers, (Ergezer and Leblebicioǧlu, 2016) have proposed a novel formula $\frac{1}{2}\bar{R}\leq R\leq \bar{R}.$ Let the matrix *K* = *A^T^A* where *A* is denoted as transform matrix, the slack variables δ_*i*_(*i* = 1, 2, 3,*n*). The paradigm of linear F-SVM is represented in (2):


(4.5) (wTK-1w)w,b,δ,Kmin21Z∑i=1nδi+ρtr(KS)

$$s.t.y_{i}\left(w^{T}x_{i}b\right)\geq 1-\delta_{i}\forall_{i}$$

$$\delta_{i}=0,i=1,2,3,\ldots ,$$

K≻0

Wu et al. (2018) solved the nonlinear classification problems by incorporating kernel PCA into linear F-SVM. The proportion of cumulative eigenvalues to the sum of all eigenvalues is set as 0.9 in the dimension selection of kernel PCA. The paradigm can be formulated as follows:


(4.6) (wTK-1w)min21Z∑i=1nδi+ρtr(KNq)

$$s.t.y_{i}\left(w^{T}f_{i}+b\right)=1-\delta_{i}\forall_{i}$$

$$\delta_{i}=0,i=1,2,3,\ldots ,$$

K≻0

where $N_{q}=\sum_{i=1}^{n}w_{i}q_{i}q_{i}^{T},q_{i}=Q^{T}\emptyset \left(x_{i}\right),Q=\left[q_{1},q_{2},q_{3},\ldots ,q_{Go}\right]$ is indicated tothe eigenvectors corresponding to the first G eigenvalues. The mapping function of kernel F-SVM that is always utilized is radial-basis-function (RBF), i.e., (*x*_*i*_,*x*_*j*_) = exp(−γ||*x*_*i*_−*x*_*j*_||^2^), where γ is the specified parameter to limit the width of the RBF (Chen et al., 2014). Between the minimization of training error and maximization of the classification margin in the paradigm, factor Z controls the trade-off (Tharwat and Hassanien, 2018). The classification accuracy differs between these two parameters. Therefore, defining the values of the parameters is essential to the performance of the SVM classifier.

### Fruit Fly Optimization Algorithm

The fruit fly optimization algorithm is based on the foraging behavior of the insect after which it is named (Pan, 2012). The main concept of the algorithm is that the insect primarily flies toward food *via* utilizing its olfactory sensory neurons: one of the groups of neurons will emit a pheromone when it is near to food. Thereafter, the fruit fly will change its direction and fly to meet its peers. Through continually updating its status and flying direction, the fruit fly will finally get nearer to the food, the position of which is the optimum solution. The algorithm will be completed if the iteration reaches maximization or the outcome is to archive the permissible accuracy. The algorithm can be split into a number of steps:

1) The position of the fruit fly is random initialization (*InitX, InitY*).
2) For each fruit fly, given a random direction and distance to hunt for food *via* its olfactory sensory neurons:

$$X_{i}=X+Randomvalue$$

$$Y_{i}=Y+Randomvalue$$

3) Due to the unknown exact location of food, the distance will be computed from the location of the fly to the origin; thereafter, the mutual distance is computed. As a result, the value will be defined as a smell concentration judgment value (*d*):

$$Dist_{i}={\left(X_{i}^{2}+Y_{i}^{2}\right)}^{1/2}$$

$$d1_{i}=\frac{1}{Dist_{i}}$$

4) to detect a better smell concentration, set the above smell concentricity judgment value into smell concentricity judgment function:

$$Smell_{i}=Function\left(n_{i}\right)$$

5) discover individuals with the raised concentricity in the population:

[bestSmell,bestIndex]=max(Smell)

6) preserve the most appropriate concentricity and an assortment of the fruit fly, and other fruit flies to that coordinates utilizing vision:

X=X(bestindex)

Y=Y(bestindex)

7) In Steps 2-5, the iterative optimization was performed. Thereafter, judge whether the concentricity is higher than that of the former level. If so, perform Step 6.

### Classification Based on FOA-F-SVM Model

**FIGURE 3:**
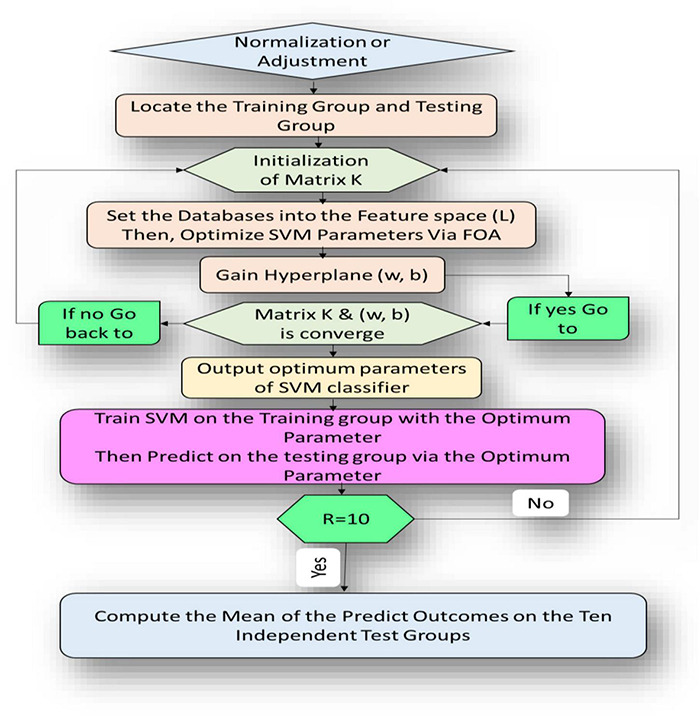
Flow diagram representation algorithm of the proposed FOA-F-SVM model.

This section introduces the main idea used in developing the newly proposed FOA-F-SVM system. In order to improve and further develop the performance accuracy of the traditional SVM model, the F-SVM for joint learning of the feature transformation and SVM classifier integrated with FOA were proposed for the analysis of alcoholism through multichannel EEG signals. As shown in Figure 3, the proposed model consists of different stages. The first five steps represent internal parameter optimization and the next five steps display the external evaluation of the classification performance. The path of the proposed model is this: tune parameters depend on the FOA, after that gain an optimum classifier. Eventually, by testing the dataset through external assessment, the performance of the classifier was measured.

The fruit fly optimization algorithm was utilized to set the parameters in the section of parameter optimization. Depending on the RBF kernel of the SVM classifier, the fruit fly’s solution was used to represent the classifier parameters Z and γ. To direct the updating of the fruit fly location, the rate of classification accuracy of the structure SVM classifier was used. The optimum solution was gained *via* the iterative optimization procedure, depending on the location. The SVM classifier was built up with the optimum parameters gained above in the external assessment section; thereafter, the eventual classification outcomes were gained on the test set *via* this classifier.

### Optimization Algorithm

In the FOA-F-SVM model, there are many unknown variables, such as in the formula (11). To solve obscure variables (matrix *K* and hyperplane (*w*,*b*)) of the FOA-F-SVM model, there are three main steps:

***(i)* Initiating *K***
Suppose the weighted covariance *N*_*q*_ performs eigenvalues decomposition, i.e., *N_*q*_ = D⋀D*^T^**, where *⋀ = *Diag*{λ_1_,_2…_, λ_*n*_}* and λ is arranged in order from highest to lowest. After algebraic computation, matrix K_0_ can be denoted as *K*_0_ = *D*_∧_^−(1/2)^*DT*. Due to *K* = *A*^T^*A*, the transformation matrix *A* can be written as *A*_0_ = ∧^−(1/4)^*DT*. Therefore, the samples are transformed into *z* = *dataset* * *A*_0_.
***(ii) Resolve hyperplane (w, b)***
This step consists of an explanation of how the FOA model is adopted to gain an optimum SVM classifier. The particular operation is that: the range of each parameter is given; thereafter, various values are randomly allocated within this range for every fruit fly. In the meantime, the fruit fly is represented in every group solution. Subsequently, find the preferable of these solutions. The finding operation includes two portions: *via* a smell search procedure, every fruit fly adjusts its position; based on the preferable fruit fly through the vision-based search procedure, the worst fruit fly in the population will be encouraged. This will then lead to obtaining a solution of the parameters *via* the iteration. Eventually, the test samples from *z* and gained optimum parameters are fed to the F-SVM prediction model.
***(iii) Resolve matrix K***
Now, having gained the SVM classifier, formula (11) can be formulated again as follows:

(4.7) fKmin(K)=(wTK-1w)21Z∑i=1nδi+ρtr(KN)

s.t.K≻0

The function is cambered and is able to be differentiated for *K*, thus, to solve *K*, the gradient-projection method was chosen. The derived function for this term is given below. Thereafter, update *K via K*_*h*1_ = *PN*(*K*_*h*_−*t*_1_∇⁡*f*(*K*_*h*_)) until *K* converges.

(4.8) $$\nabla f\left(K\right)=-\frac{1}{2}K^{-1}ww^{T}K^{-1}+\rho N$$

**(iv)** From all the illustrations and explanations above, it is clear that the matrix *K* is a significant parameter in the FOA-F-SVM. Only *via* initializing *K*, it can transform the dataset into a new feature space. Thereafter, an SVM classifier is gained *via* optimizing parameters through FOA. Eventually, an optimal classifier is gained by constantly updating *K*.

## Performance Evaluation Methods

It is important to evaluate the performance of any classification or detection system. A set of methods was used to assess the performance of the alcoholism classification and detection system based on the proposed FOA-F-SVM technique, as described below:

(a) Accuracy (Acc.) is a degree of proximity of a measured or calculated quantity to its actual (true) value. The term accuracy is utilized to assess the performance of the SVM method depending on the formula as given below:

(4.9) Acc.=(TPTN)/(TPTNFPFN)

(b) Sensitivity (Sen.) is a statistical measure of the performance of a binary classification test used to measure the rate of the real positive prediction. This is defined as follows:

(4.10) Sen.=TP/(TPFN)

(c) Specificity (Spe.) is utilized to measure the proportion of the real negative predication and is defined as follows:

(4.11) Spe.=TN/(TNFP)

(d) Predictive positive value (PPV) is defined as the rate of positives that correspond to the presence of the condition described *via* the formula as below:

(4.12) PPV=TP/(TPFP)

(e) Predictive negative value (PNV) is the ratio of negatives that correspond to the absence of the condition and is defined as follows:

(4.13) PNV=TN/(TNFN)

## Experimental Results

To conduct the simulation effectively, the same number of iterations and the same population size were set for particle swarm optimization (PSO), genetic algorithm (GA), and FOA. According to our preliminary experiment, when the number of maximum iteration and population size are, respectively, set as 100 and 20, the methods involved result in satisfactory classification performance. Furthermore, in the experiment, parameter *Z* is in range *Z* ∈ {2^−10,1,20^}, parameter *g* is set *asg* ∈ {2^20,1,10^}. The parameters of each model are as follows: for FOA-F-SVM, the *x* and *y* are denoted to initialize the location of fruit fly and the search direction *ax*,*bx*,*ay*,*andby* are set as 10, 20, 20, and 10, respectively, in the distance function. For PSO-SVM, the maximum velocity is 0.5 times the maximum parameter *Z*. The learning factors *Z*1,*Z*2 were set as 1.6, 1.5, and the intermediate variable *w* was set as 1 in the updating velocity function and updating location function. All experiments were carried out on a desktop computer with a CPU (2.30 GHz) and 8.00 GB RAM under the MATLAB 2020 programming environment.

The experimental EEG data used to assist the proposed model were obtained from the University of California, Irvine Knowledge Discovery in Databases Archive UCI KDD. The EEG signals were collected from 122 participants, and each subject performed 120 trials with three types of stimuli (Tao et al., 2020). The recordings were obtained from 61 channel EEG signals, two EOG channels, and one reference electrode. There are three datasets, named SMNI_CMI_- TRAIN, SMNI_CMI_TEST, and FULL, respectively. In this study, only the first two databases were utilized because the full datasets contain a few all-zero recordings. There were 600 recorded files in SMNI_CMI_TRAIN, with each recording containing the signals from 64 electrodes caps. The 64 electrodes are *FC_4_, FC_3_, C_6_, C_5_, F_2_, F_1_, TP_8_, TP_7_, AFZ, CP_3_, CP_4_, P_5_, P_6_, C_1_, C_2_, PO_7_, FP_1_, FP_2_, F_7_, F_8_, AF_1_, AF_2_, FZ, F_4_, F_3_, FC_6_, FC_5_, FC_2_, FC_1_, T_8_, T_7_, CZ, C_3_, C_4_, CP_5_, CP_6_, CP_1_, CP_2_, P_3_, P_4_, PZ, P_8_, P_7_, PO_2_, PO_1_, O_2_, O_1_, X, AF_7_, AF_8_, F_5_, F_6_, FT_7_, FT_8_, FPZ, PO_8_, FCZ, POZ, OZ, P_2_, P_1_, CPZ, nd*, and *Y*. The electrodes *X* and *Y* are EOG signals, and *nd* is reference electrode. The EOG and *nd* were removed in our analysis. However, the features were extracted from 61 channels.

### Features Selection Using KST

In this section, six experiments were conducted to select the most powerful features to classify EEG signals.

***In the first experiment***, 11 channels were tested (*AF_1_, AF_2_, AF_7_, AF_8_, AFZ, C_1_, C_2_, C_3_, C_4_, C_5_*, and *C*_6_) to determine whether these channels were adequate to analyze the alcoholism signals. Table 1 reports the results of feature selection using KST. Based on statistical analysis, the results showed that using these channels could explain 60% of the data.

**TABLE 1:** Feature set outcome of Experiment No. 1.

Features	Testing	Training	Compared with the *p*-values
		
	Controlled vs. Alcohol	Controlled vs. Alcohol	
Mean	0.1088	0.2003	Rejected
Max	0.46	0.342	Rejected
Med	0.0017	2.9480 × 10^–09^	Accepted
Min	0.011	0.02	Accepted
Mod	0.011	0.02	Accepted
Range	1.7552 × 10^–05^	0.034	Accepted
Skew	0.1088	0.94	Rejected
Kur	0.1	0.93	Rejected
Std.	2.0212 × 10^–04^	0.01088	Accepted
Var.	1.7552 × 10^–05^	0.02003	Accepted

***In the second experiment***, the channels *AF_8_, C_1_, C_2_, C_3_, C_4_, CP_1_, CP_5_, CP_6_, FC_5_, FT_7_, P_8_, PO_8_*, and *P* were utilized in the second experiment below. The outcomes indicate that the acceptance rate was high, reaching 90%, which means that the signal in these channels was suitable for detecting the EEG signals. Table 2 reports the obtained results.

**TABLE 2:** Feature set outcome of Experiment No. 2.

Features	Testing	Training	Compared with the *p*-values
		
	Controlled vs. Alcohol	Controlled vs. Alcohol	
Mean	5.5870 × 10^–08^	0.02585	Accepted
Max	2.0480 × 10^–09^	0.00455	Accepted
Med	1.7973 × 10^–14^	3.5202 × 10^–10^	Accepted
Min	1.4977 × 10^–13^	0.00165	Accepted
Mod	1.4977 × 10^–13^	0.00165	Accepted
Range	2.0480 × 10^–09^	2.6199 × 10^–07^	Accepted
Skew	0.10875	0.935	Rejected
Kur	0.045	6.1578 × 10^–04^	Accepted
Std.	0.00465	0.045	Accepted
Var.	1.1088 × 10^–08^	0.00165	Accepted

***In the third experiment***, the number of channels tested was 23. The success rate was 70%. The channels were *CP_1_, CP_2_, CP_3_, CP_4_, CP_5_, CP_6_, CPZ, CZ, F_1_, F_2_, F_3_, F_4_, F_5_, F_6_, F_7_, F_8_, FC_1_, FC_2_, FC_3_, FC_4_, FC_5_, FC_6_*, and *FCZ*. Table 3 reports the results of experiment 3.

**TABLE 3:** Feature set outcome of Experiment No. 3.

Features	Testing	Training	Compared with the *p*-values
		
	Controlled vs Alcohol	Controlled vs Alcohol	
Mean	0.055	0.3420	Rejected
Max	0.0259	0.0017	Accepted
Med	0.0113	1.7552 × 10^–05^	Accepted
Min	1.1615 × 10^–12^	5.6313 × 10^–11^	Accepted
Mod	1.1615 × 10^–12^	5.6313 × 10^–11^	Accepted
Range	0.05	0.0113	Accepted
Skew	0.2003	0.76	Rejected
Kur	0.5372	0.9360	Rejected
Std.	6.1578 × 10^–04^	0.011	Accepted
Var.	0.0113	0.002	Accepted

***In the fourth experiment***, the acceptance rate was 50%. A total of twenty-eight channels passed the test in this experiment. The channels used in this experiment were *FP_1_, FP_2_, FPZ, FT_7_, FT_8_, FZ, O_1_, O_2_, OZ, P_1_, P_2_, P_3_, P_4_, P_5_, P_6_, P_7_, P_8_, PO_1_, PO_2_, PO_7_, PO_8_, POZ, PZ, S_1_, T_7_, T_8_, TP_7_*, and *TP*_8_ (Table 4).

**TABLE 4:** Feature set outcome of Experiment No. 4.

Features	Testing	Training	Compared with the *p*-values
		
	Controlled vs Alcohol	Controlled vs Alcohol	
Mean	0.34	0.2	Rejected
Max	0.53	0.20	Rejected
Med	0.002	0.005	Accepted
Min	0.06	0.2003	Rejected
Mod	0.06	0.2003	Rejected
Range	0.012	0.0017	Accepted
Skew	0.8	0.54	Rejected
Kur	0.026	0.0259	Accepted
Std.	0.005	0.0046	Accepted
Var.	6.1578 × 10^–04^	0.005	Accepted

***In the fifth experiment***, the channels *AF_1_, AF_2_, AF_7_, AF_8_, AFZ, FP_1_, FP_2_, FPZ, FT_7_, FT_8_, P_1_, P_2_, P_3_, P_4_, P_5_, P_6_, P_6_, P_7_, P_8_, PO_1_, PO_2_, PO_7_, PO_8_, POZ, F_1_, F_2_, F_3_, F_4_, F_5_, F_6_, F_7_, F_8_, T_7_, T_8_, TP_7_*, and *TP*_8_ were used in this experiment. The acceptance rate was very low, that is, 40%. This indicates that the channels used were not valid for classification (Table 5).

**TABLE 5:** Feature set outcome of Experiment No. 5.

Features	Testing	Training	Compared with the *p*-values
		
	Controlled vs Alcohol	Controlled vs Alcohol	
Mean	6.1740 × 10^–05^	0.012	Accepted
Max	2.0212 × 10^–04^	0.109	Accepted
Med	1.7973 × 10^–14^	0.03	Accepted
Min	0.34	0.9	Rejected
Mod	0.34	0.9	Rejected
Range	2.0212 × 10^–04^	0.005	Accepted
Skew	0.55	0.54	Rejected
Kur	0.93	0.4	Rejected
Std.	0.76	0.46	Rejected
Var.	0.1088	0.01	Rejected

***In the sixth experiment***, the results obtained from Experiment No. 6 indicate that the use of 61 channels was efficient in the analysis. They could, thus, be used to classify EEG signals. The 61 channels were as follows: *FC_4_, FC_3_, C_6_, C_5_, F_2_, F_1_, TP_8_, TP_7_, AFZ, CP_3_, CP_4_, P_5_, P_6_, C_1_, C_2_, PO_7_, FP_1_, FP_2_, F_7_, F_8_, AF_1_, AF_2_, FZ, F_4_, F_3_, FC_6_, FC_5_, FC_2_, FC_1_, T_8_, T_7_, CZ, C_3_, C_4_, CP_5_, CP_6_, CP_1_, CP_2_, P_3_, P_4_, PZ, P_8_, P_7_, PO_2_, PO_1_, O_2_, O_1_, AF_7_, AF_8_, F_5_, F_6_, FT_7_, FT_8_, FPZ, PO_8_, FCZ, POZ, OZ, P_2_, P_1_, CPZ* (Table 6).

**TABLE 6:** Feature set outcome of Experiment No. 6.

Features	Testing	Training	Compared with the *p*-values
		
	Controlled vs Alcohol	Controlled vs Alcohol	
Mean	0.045	0.0446	Accepted
Max	0.3420	0.1088	Rejected
Med	6.1740 × 10^–05^	1.7973 × 10^–14^	Accepted
Min	1.4977 × 10^–13^	0.026	Accepted
Mod	1.4977 × 10^–13^	0.026	Accepted
Range	0.011	0.03	Accepted
Skew	0.1	0.76	Rejected
Kur	0.046	0.034	Accepted
Std.	0.00238	0.01	Accepted
Var.	0.0476	0.02	Accepted

As a result, with the highest acceptance rates, the second and sixth experiments performed the best. The last group of features utilized to identify each pair of EEG groups (Controlled *vs*. Alcoholic) were *[Mean, Median, Minimum, Mode, Range, Kurtosis, SD, and Variance].* Therefore, by conducting a number of experiments, we were able to thoroughly investigate the feature selection in order to select the most effective feature set to recognize EEG groups.

### Evaluating the Performance of the Proposed FOA-F-SVM Model

To evaluate the performance of the FOA-F-SVM in alcoholic EEG signals, a comparison was made with SVM, PSO-SVM, GA-SVM, and F-SVM. Table 7 shows the average results of the comparison among the FOA-F-SVM, PSO-SVM, GA-SVM, F-SVM, and SVM. Based on the results, the performance of the FOA-F-SVM attains higher classification accuracy than other approaches. However, the PSO-SVM and GA-SVM scored the second highest results, and they outperformed the basic SVM. These research findings indicate that tuning parameters were important in improving classification accuracy of EEG signals. In addition, the classification accuracy obtained by the F-SVM is higher than the basic SVM.

**TABLE 7:** Classification accuracy of the comparison among FOA-F-SV, PSO-SVM, GA-SVM, F-SVM, and SVM.

Approach	Accuracy	Sensitivity	Specificity
FOA-F-SVM	99.2%	98.4%	98.5%
PSO-SVM	95.5%	94.3%	95.9%
GA-SVM	96.5%	95.2%	95.3%
F-SVM	92.5%	91.7%	92.4%
SVM	85.5%	86.2%	84.6%

**FIGURE 4:**
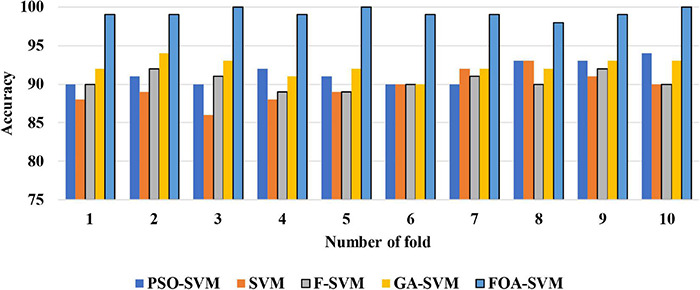
The detailed classification accuracy of 10 folds.

Figure 4 shows the detailed classification accuracy of 10 runs, as well the results of FOA-F-SVM, which are up to 98%, while the results of PSO-SVM and GA-SVM are distributed in the range from 90 to 94%. While the F-SVM and SVM gained a rate of accuracy from 86 to 93%. As a result, it can be observed that the FOA-F-SVM obtained the highest accuracy on each run and the best value is 100%. However, because of the robustness of the proposed method, the average result is the highest with 99.2%.

### Channel Selection Based on Classification Accuracy

**FIGURE 5:**
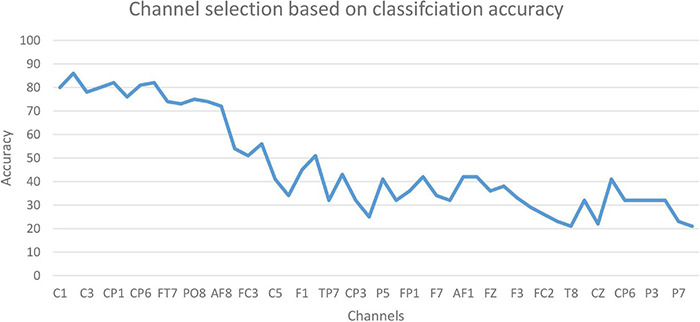
The accuracy based on EEG channels.

The accuracy of the proposed model based on 61-channel EEG signals is shown in Figure 5. In this experiment, the features were extracted from each channel and forwarded to the proposed model. The results show that not all channels yielded high classification accuracy. As a result, 13 optimal channels, including *AF_8_, C_1_, C_2_, C_3_, C_4_, CP_1_, CP_5_, CP_6_, FC_5_, FT_7_, P_8_, PO_8_, PZ*, were selected and used to classify EEG signals as shown in Figure 5.

The results in Figure 5 are compatible with the results obtained by statistical metrics in the feature selection and enhanced the results (not all channels gave high classification accuracy). The present study thus demonstrates the ability of the proposed model to assess alcoholic EEG signals from multichannel EEG signals. The extracted features from electrodes C1, C3, and FC5 were found to be significantly effective in classifying EEG signals: an accuracy of 87.6 % was achieved. In addition, it was found that when the 13 channels were used to extract the features, the classification accuracy was close to the whole 61-channel performance. Table 8 presents the classification accuracy based on the number of channels.

**TABLE 8:** Classification accuracy based on the number channels.

Channel No.	Accuracy	Sensitivity	Specificity
C_1_, C_3_ and FC_5_	85.6%	83.8%	82.4%
AF_8_, C_1_, C_2_, C_3_, C_4_, CP_1_, CP_5_, CP_6_, FC_5_, FT_7_, P_8_, PO_8_, P	99.4%	98.7%	99.1%
All 61 channels	99.5%	98.3%	99.2%

## Discussion

This study carried out an analysis of EEG signals to detect the prevalence and health effects of alcoholism from multichannel EEG signals. We integrated the CT with BS, CT-BS, to reduce the dimensionality of EEG signals. Then, the covariance matrix with its eigenvalues was applied to investigate the EEG signals, and to extract the important features. Arithmetic operators based on the KST technique were utilized to remove the noisy features from the obtained features set. The FOA-F-SVM was proposed to classify the EEG signals. The proposed FOA-F-SVM classification mode was compared with different methods such as SVM, PSO-SVM, GA-SVM, and F-SVM. In this section, we summarized the following main findings:

(1) The novelty of this article lies in the utilization of CT and BS (CT-BS) coupled with the covariance matrix for feature extraction. It has been shown that the low dimensionality of EEG signals achieved by CT-BS can efficiently improve the classification rate. In comparison to other dimension-reduction techniques such as linear discriminate analysis (LDA) and PCA, the experimental results indicate that CT-BS performs better than PCA and LDA, and the classification rate of the FOA-F-SVM was increased with CT-BS by more than 9%. Table 9 reports the classification rate based on dimension reduction techniques.

**TABLE 9:** Classification rate based on different features reduction algorithms.

Technique \metrics	Accuracy	Sensitivity	Specificity
PCA with OA-F-SVM	89.1	90.2	88.2
LDA with OA-F-SVM	87.4	85.3	86.3
CT-BS with OA-F-SVM	99.2	98.1	99.3

**FIGURE 6:**
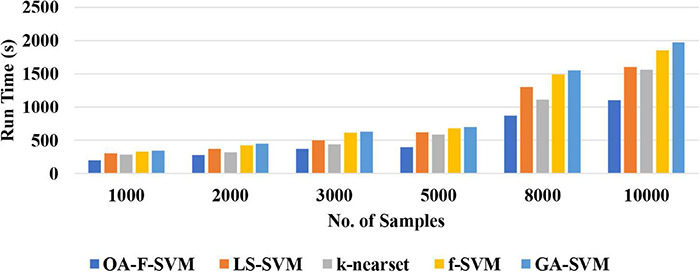
A Comparison of run time among the proposed model with other.

**FIGURE 7:**
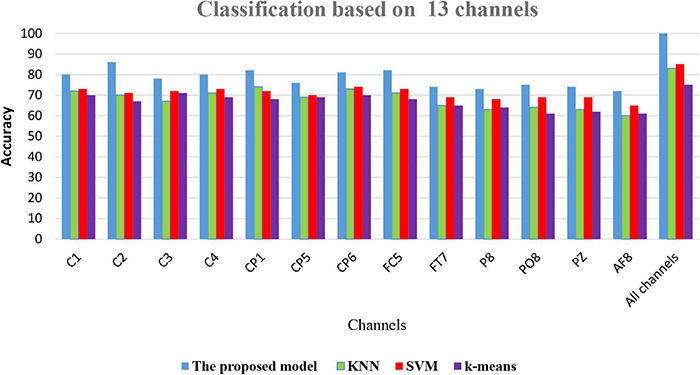
A Comparison among the proposed model with SVM, *k*-means, and KNN.

(2) The proposed approach is a simple classification method for the identification of normal *versus* alcoholic EEG signals. The complexity of the proposed method was tested using a different number of samples. The results of the simulation showed that the proposed method achieved a better performance among traditional classification algorithms with acceptable time consumption. Therefore, this method could be a practical and feasible model for a real-time brain–computer interface (BCI) system. Figure 6 reports the run time of the proposed classification model compared with LS-SVM, k-nearest, f-SVM, and GA-SVM. It can be noticed that the proposed model is faster than LS-SVM, k-nearest, f-SVM, and GA-SVM.

(3) The proposed model is still at the experimental stage. Larger datasets are required to make further validation of this model before it could be utilized as a tool in real-time applications.
(4) In this article, a small EEG dataset was used to evaluate the proposed model. The next work will focus on the use of a large EEG dataset such as EEG sleep stages, aesthetic EEG data, to analyze the performance of the proposed model under a huge dataset. This can guide us to improve the effectiveness of the proposed model.
(5) Although the CT-BS technique improved the performance of the classification model, it took more time than the PCA and LDA. In the future, we will work on how to reduce the complexity time of the CT-BS model.
(6) Comparison of classification accuracy of the proposed model FOA-F-SVM with KNN, k-means, and SVM: In this experiment, on the performance of the proposed model, FOA-F-SVM based on 13 EEG channels was reported. For further verification and to reach the highest level of reliability, the results were compared with KNN, k-means, and SVM. To the best of our knowledge after extensive research, this is the first time the FOA-F-SVM model has been proposed and applied to the analysis and detection of alcoholism EEG signals. The results showed that compared to other algorithms, the proposed model FOA-F-SVM has promising performance that can be adopted as a classification technique of alcoholism EEG signals. The database SMNI_CMI_TRAIN was used for the training, and the database SMNI_CMI_TEST was utilized for the testing set. To show clearly the classification results based on the 13 selected channels, Figure 7 depicts the accuracy of the proposed model FOA-F-SVM with KNN, k-means, and SVM. The proposed model outperformed KNN, k-means, and SVM over all the 13 channels. In addition, the proposed model achieves 99% when all channels are used for the classification of EEG signals.

(7) Many studies were focused on finding a system that could be utilized for the automated detection of alcoholism EEG signals to estimate the effect of treatment and help significantly with clinical diagnosis. In this point, we shall review some of the previous studies that used the same data as this work did; for each, we shall provide a comparison of results. The identification of nonlinear features such as SAMENT, APPENT, largest Lyapunov exponent (LLE), and higher-order spectra (HOS) with LS-SVM classifier was used by Acharya et al. (2012), who obtained an average classification accuracy of 91.7%. However, the classification accuracy that is achieved by the proposed model is significantly higher than that of Acharya et al. (2012). Another group of researchers (Faust et al., 2013b) has improved an automated system utilizing wavelet packet-based energy measures with the KNN classifier; this method achieved a classification accuracy of 95.8%, which is less than the rate obtained by the proposed model.

A study by Patidar et al. (2017) suggested an automated system for the diagnosis of alcoholism. The study utilised TQWT to decompose EEG signals into various bands (SBS). Compared to the results obtained by the proposed method, the model of Patidar et al. (2017) obtained a classification accuracy of 97.02%, which is, again, less than our classification accuracy of 99%. For the detection of alcoholic-related changes in EEG signals, (Pan, 2012) have proposed the use of HOS cumulants-based features. Based on the fuzzy Sugeno classifier (FSC), the investigators achieved a classification accuracy of 92.4%, which is considerably less than the 99% obtained in the present work. Finally, the largest Lyapunov exponent (LLE), entropies, correlation dimension (CD), and Hurst exponent (H) were proposed by (Kannathal et al., 2005) to obtain the features for detecting alcoholism from EEG signals: the rate of accuracy was 90%, which is considerably less than the classification accuracy achieved by the model proposed in this study. Anuragi et al. (Anuragi and Sisodia, 2020) proposed an adaptive filtering model to extract time–frequency-domain characteristics from Hilbert–Huang transform. LS-SVM and KNN were used to classify the extracted features into alcoholic and normal signals. Bavkar et al., 2021) also applied empirical mode decomposition to classify alcoholic EEG signals. The extracted features using empirical mode decomposing were sent to the KNN classifier.

The results in Table 10 show that the method proposed was superior to other studies and obtained a higher level of accuracy. After conducting many experiments and various types of comparisons, it has become clear that the proposed CT-BS-OFA-F-SVM model has a promising future in analyzing and classifying EEG signals with a high rate of accuracy. It was also noted that most of the previous studies were working on developing one part of the analysis, whereas, in this study, the focus was on most of the analysis steps.

**TABLE 10:** Comparison with existing methods using the same database.

Authors	Features/techniques	Analysis	Accuracy
Acharya et al. (2012)	APPENT, SAMENT, LLE	SVM	91.7%
Faust et al. (2013b)	WPT, energy measures	KNN	95.8%
Patidar et al. (2017)	TQWT, CE	LS-SVM	97.02%
Faust et al. (2013a)	HOS cumulants	FSC	92.4%
Kannathal et al. (2005)	CD, LLE, entropy, H	Unique ranges	90%
Anuragi and Sisodia (2020)	Empirical wavelet transform	LS-SVM, KNN	98.75%
Bavkar et al. (2021)	Empirical Mode Decomposition	KNN	93.87%
**The proposed model**	**CT-BS-Cov-Eig**	**FOA-F-SVM**	**99%**

## Conclusion

Accurate detection algorithms can be used effectively to help clinical research as a fast, reliable, and easy-to-use tool in the diagnosis and monitoring of neurological disorders and in alcoholism. We developed an effective method that was designed for sampling by integrating CT and BS (CT-BS) in one phase. To detect and analyze abnormalities in the EEG signal, the eigenvalues of the covariance matrix were investigated utilizing a statistical method that extracted ten statistical features from the eigenvalues of the covariance matrix. To classify EEG signals, the FOA-F-SVM was proposed to detect and analyze multichannel EEG signals. The proposed model was compared to previous studies, and the results showed that the proposed model was superior, with a high accuracy rate of 99%.

The acquired results clearly illustrate the superior performance of the proposed CT-BS model coupled with FOA-F-SVM to the existing state-of-the-art methods. The proposed model can be used to assist neurologists and other medical specialists in the precise diagnosis of alcoholism EEG signals. Future studies may investigate the improvement of the performance of the proposed model by decreasing the number of features used in this initial study. Also, because there is a great similarity between the results of feature selection and the results of channel selection, the possibility of proposing and implementing feature selection methods will be studied to find the optimal channels. Furthermore, with regard to the few numbers of studies focused on designing feature extraction as well as a detection model for the reliable diagnosis of alcoholism EEG signals, there is a need for further research in this area.

## Data Availability Statement

The original contributions presented in the study are publicly available. This data can be found here: https://kdd.ics.uci.edu/databases/eeg/eeg.html.

## Author Contributions

SA: conceptualization, methodology, software, validation, data curation, formal analysis, and writing – review and editing. MD: methodology, resources, software, and validation. RD: investigation, methodology, resources, software, validation, visualization, and writing - review and editing. JG: writing - review and editing and investigation. All authors contributed to the article and approved the submitted version.

## Conflict of Interest

The authors declare that the research was conducted in the absence of any commercial or financial relationships that could be construed as a potential conflict of interest.

## Publisher’s Note

All claims expressed in this article are solely those of the authors and do not necessarily represent those of their affiliated organizations, or those of the publisher, the editors and the reviewers. Any product that may be evaluated in this article, or claim that may be made by its manufacturer, is not guaranteed or endorsed by the publisher.
